# Regressed Papillary Thyroid Carcinoma with Anaplastic Transformation into Lymph Node Metastasis: Case Report with Review of the Literature

**DOI:** 10.3390/diagnostics15050523

**Published:** 2025-02-21

**Authors:** Bozidar Kovacevic, Bojana Rancic, Sasa Jovic, Snezana Cerovic, Vesna Skuletic, Jelena Karajovic, Milka Gardasevic, Gordana Supic, Kennichi Kakudo

**Affiliations:** 1 Institute of Pathology and Forensic Medicine, Military Medical Academy, 11000 Belgrade, Serbia; bojana.jovanovic.vma@gmail.com (B.R.); cerovics@gmail.com (S.C.); vesna.skuletic@gmail.com (V.S.); 2 Medical Faculty of the Military Medical Academy, University of Defense, 11000 Belgrade, Serbia; sasajovic71@icloud.com (S.J.); gogasupic@gmail.com (G.S.); 3 Clinic of Maxillofacial Surgery, Military Medical Academy, 11000 Belgrade, Serbia; gardmilka16@gmail.com; 4 Clinic of Endocrinology, Military Medical Academy, 11000 Belgrade, Serbia; karajovicjelena@gmail.com; 5 Institute for Medical Research, Military Medical Academy, 11000 Belgrade, Serbia; 6 Department of Pathology, Wakayama University Medical School, Wakayama 641-0096, Japan; kakudo@thyroid.jp

**Keywords:** spontaneous tumor regression, papillary thyroid carcinoma, anaplastic transformation, anaplastic thyroid carcinoma

## Abstract

**Background**: Small papillary thyroid carcinomas with the largest dimension of 10 mm are slow-growing and self-limiting tumors, most of which have no potential for progression, rarely becoming clinically evident carcinomas or undergoing regressive changes. Their anaplastic transformation, primarily in the thyroid gland or into lymph node metastasis, is extremely rare. **Case Presentation**: A 66-year-old female patient was admitted to our hospital for diagnostics and treatment of a large tumor on the left neck side. Preoperative imaging and cytological findings of the neck tumor suggested metastasis of papillary thyroid carcinoma. Total thyroidectomy and metastasectomy were performed. In the final diagnosis, anaplastic transformation of the papillary thyroid carcinoma’s metastasis in the neck was confirmed. Opposite to advanced dedifferentiation of metastasis, primary tumor foci in the thyroid were regressed and replaced with sclerosis and microcalcification. The synchronous co-occurrence of incidental primary thyroid carcinoma and anaplastic thyroid carcinoma originating from ectopic cervical thyroid tissue was considered diagnostically. **Conclusions**: The case highlights the necessity of regular monitoring of the thyroid and neck lymph nodes for patients under active surveillance, including those with small calcified tumor foci. This paper also comprehensively reviews the existing literature on this topic.

## 1. Introduction

Spontaneous tumor regression (STR) is a rare, natural, well-documented process. It implies partial or complete tumor disappearance without previously applied therapy [1,2]. STR has been reported in various types of neoplasms such as renal cell carcinoma, melanoma, neuroblastoma, testicular germ cell tumors, lymphoma, and pancreatic and thyroid carcinoma [1,2,3,4]. It is assumed that the basis of STR is primarily immune-mediated. An activated immune system restricts tumor growth by creating an antitumor microenvironment and/or acting directly on tumor cells through the induction of apoptosis [1,2]. Papillary thyroid cancer (PTC) is the most common primary thyroid cancer, with a favorable clinical course and a ten-year survival rate of more than 90%. The 10-year survival rate of patients with subcentimetric PTC (s-PTC) is even better, with a mortality rate of nearly 0.1% [5]. s-PTCs, in previous tumor classifications defined as papillary thyroid microcarcinomas, are slow-growing and self-limiting tumors, most of which have no potential for progression, rarely becoming clinically evident carcinomas or even undergoing regressive changes [5,6,7]. On the contrary, anaplastic thyroid carcinoma (ATC) is a rapidly growing, aggressive, and lethal neoplasm, with disease-specific mortality coming to 100%. It accounts for less than 2% of primary thyroid malignancies. ATC often arises from preexisting well-differentiated follicular-cell-derived carcinomas through a multi-step process that usually includes additional mutations in the *p53* and *TERT* promoter genes [5,6]. Dedifferentiation of PTC to ATC can occur primarily in the thyroid or rarely by dedifferentiation of local or distant metastasis [8,9]. Dedifferentiation and anaplastic transformation of primary s-PTCs in the thyroid gland or their metastases are sporadically reported in the literature [10,11]. They are associated with an unfavorable clinical course and deathly outcome. We present a unique case of s-PTC in complete regression with anaplastic transformation of neck lymph node metastasis, along with a literature review.

## 2. Case Presentation

**Figure 1:**
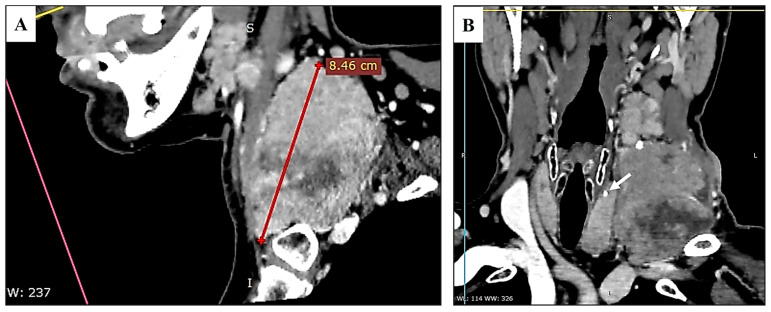
Sagittal MSCT of the neck shows a large tumor with hypodense areas of necrosis (**A**). The tumor occupied levels III, IV, and Va/Vb, infiltrated the skin, and compressed the thyroid. Thyroid tissue discloses heterodensity and foci of microcalcification (arrow) in the left lobe (**B**).

A 66-year-old female patient was admitted to our hospital for the diagnosis and treatment of a tumor on the left side of her neck. The tumor was infiltrating the skin of the supraclavicular region and extending into the chest. The patient has a history of Hashimoto’s thyroiditis and has been on 100 mg of levothyroxine for the past ten years. However, she did not attend regular follow-up check-ups to monitor her condition. She had been aware of the neck lesion for three years, and over the past two months, it had shown progressive growth. A multi-slice computed tomography (MSCT) scan with contrast of the head, neck, chest, abdomen, and pelvis was performed. The MSCT revealed a tumor mass, likely a conglomeration of lymph nodes in the left jugular region, measuring 85 × 75 mm (Figure 1).

**Figure 2:**
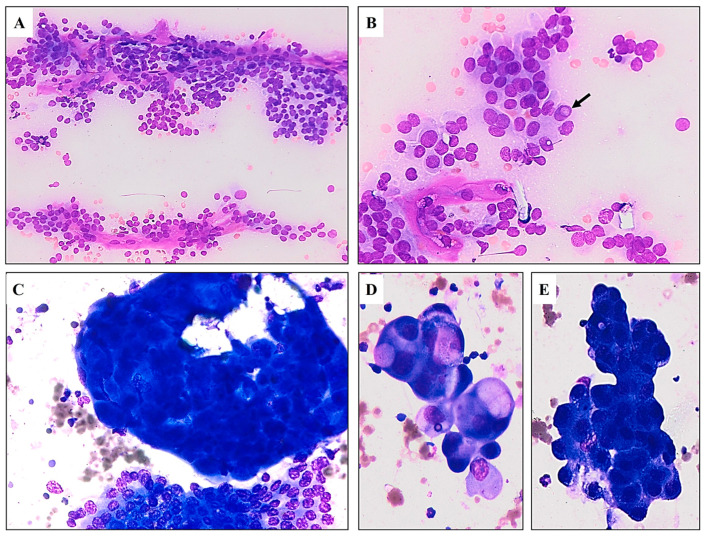
Cytological sample of the neck tumor. (**A**) Highly cellular aspirate comprises papillary structures with fibrovascular cores. Loss of cellular polarity and cohesiveness is evident (MGG*, ×10). (**B**) The nuclei of tumor cells are enlarged with irregular contours and finely textured chromatin. Occasionally, a nuclear pseudoinclusion is present (arrow) (MGG, ×20). (**C**) Two distinct cellular components are observed. The upper part of the image displays a large group of atypical cells characterized by hyperchromatic nuclei. In contrast, the lower part shows a cluster of more atypical PTC cells featuring nuclear grooves (MGG, ×20). (**D**,**E**) Groups of atypical cells with pleomorphic nuclei and conspicuous nucleoli (MGG, ×40). * May–Grunwald–Giemsa stain.

Microscopically examined cytological specimens from the neck tumor disclosed rich cellularity, with many groups of follicular cells in different architectural arrangements. The presence of papillary formation, pseudopapillary fragments, sheets, single-layered groups of cells, and the occasional microfollicle was detected. Tumor cell nuclei were enlarged and hypochromatic with grooves and pseudoinclusions. There were also groups of cells with moderately abundant basophilic cytoplasm, eccentrically placed nuclei, and conspicuous nucleoli. The specimen contained extensive cellular detritus and mixed inflammatory infiltrate with numerous macrophages and neutrophils. The cytological finding was consistent with dedifferentiated PTC with high-grade features (Figure 2) [12,13].

Total thyroidectomy with radical lymphadenectomy from the left neck side was indicated. During surgery, significant bleeding from the neck tumor required only a reduction of the lesion and total thyroidectomy. Intraoperatively, the thyroid was found to be unattached and separate from the neck tumor.

**Figure 3:**
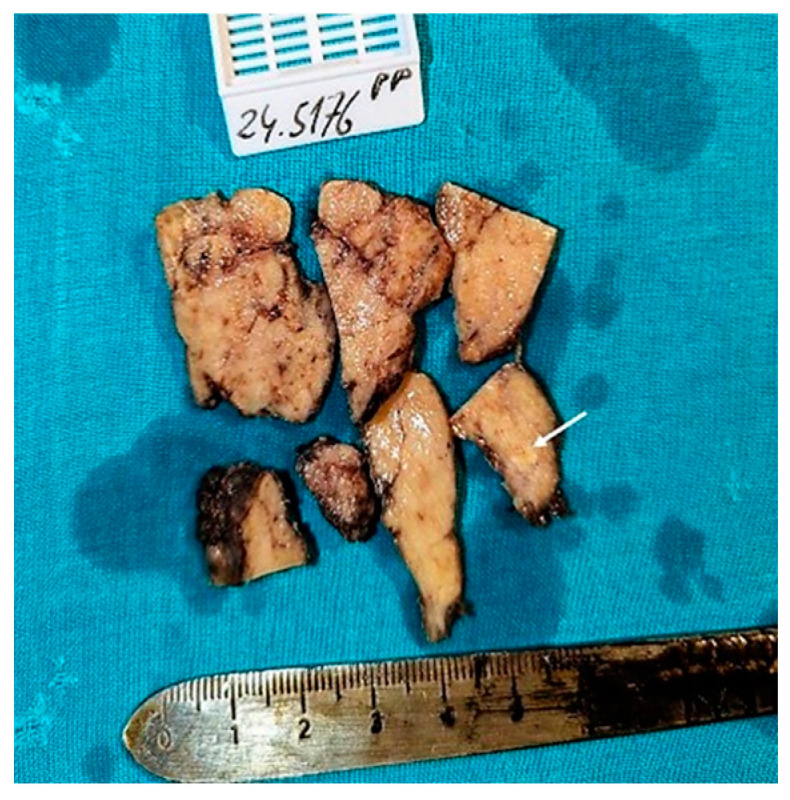
The gross appearance of the rest of the thyroid after initial processing. The tissue is grayish-white and exhibits marked lobularity. A suspected area of fibrosis with a gritty surface is noted (arrow).

Grossly, the thyroid gland weighed 18.5 g, the largest dimension of the left lobe was 43 mm, and that of the right lobe was up to 45 mm. The continuity of the thyroid surface was preserved. In the left lobe, there was a subcapsular area of sclerosis with a diameter of up to 4 mm, and in the central part of the gland, there was a 3 mm area with a fine-grained appearance and microcalcifications. The whole thyroid was processed and submitted for microscopic examination through additional sections. Figure 3 shows the macroscopic appearance of the thyroid. The removed part of the neck tumor was submitted in many fragments of various sizes with diameters reaching up to 20 mm. Necrosis dominated the specimens; the vital tumor tissue was whitish-yellow with a soft consistency. Fragments of the skeletal muscle were also present.

**Figure 4:**
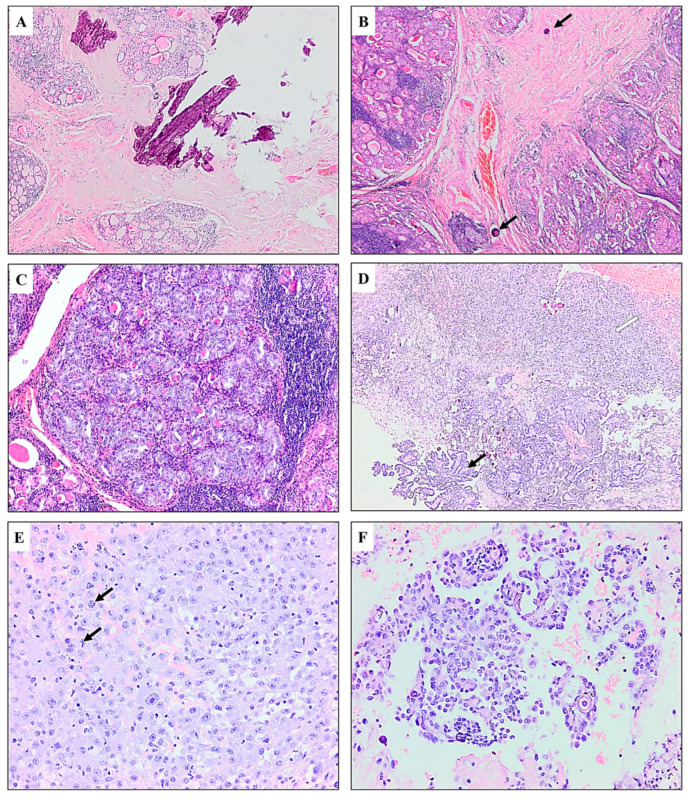
(**A**) Sclerotic area of regressed PTC with calcification (H&E*, ×5). (**B**) The periphery of the scar with psammoma bodies—arrows (H&E, ×10). (**C**) Microscopic foci of PTC in the setting of Hashimoto’s thyroiditis (H&E, ×20). (**D**) Neck metastasis containing PTC (black arrow) in continuity with epithelioid and spindle cell anaplastic areas (white arrow) (H&E, ×5). (**E**) Large anaplastic epithelioid cells have indistinct borders, basophilic cytoplasm, and pleomorphic nuclei with prominent nucleoli. Mitoses are evident (arrows) (H&E, ×20). (**F**) Hobnail subtype of PTC with loss of cellular polarity and cohesiveness (H&E, ×20). * Hematoxylin and eosin stain.

Microscopically, two stellate sclerotic areas were present in the left thyroid lobe, measuring 3 mm and 4 mm with microcalcifications and psammoma bodies. A few psammoma bodies were also noticed in the vicinity of the scars. Two separated submillimeter foci of PTC with follicular morphology were also detected. The rest of the thyroid disclosed histologic features of classic Hashimoto’s thyroiditis with accentuated gland lobulation and a pseudo-nodular appearance. Microscopic examination of the neck tumor tissue revealed two distinct morphological components. The first component included parts of the conventional subtypes of PTC and parts of PTC with loss of cellular polarity and cohesiveness with hobnail cell features [12]. The second component displayed an anaplastic feature comprising polygonal pleomorphic cells with basophilic cytoplasm and irregularly distributed spindle cells with high mitotic activity. The necrosis was massive. Figure 4 shows the microscopic appearance of the thyroid and the neck tumor.

**Figure 5:**
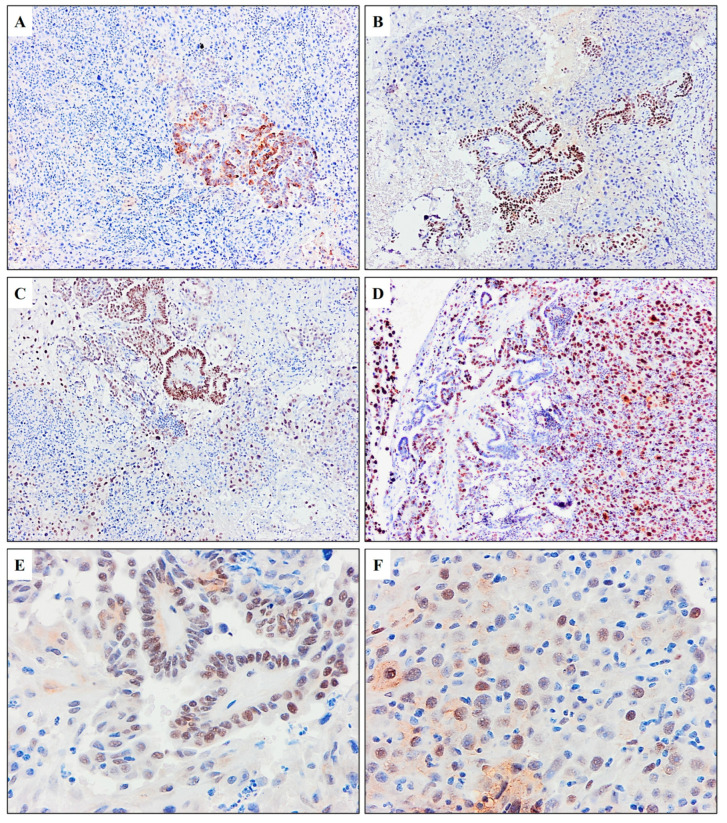
Immunohistochemical analysis of the neck metastasis. (**A**) Loss of immunohistochemical positivity for thyroglobulin (×20) and (**B**) TTF1 (×10) in tumor cells of anaplastic areas with retained positivity in parts of the PTC. (**C**) The tumor cells display nuclear positivity for PAX8 in both the anaplastic and PTC areas. (**D**) Ki67 positivity is observed in over 80% of anaplastic cells and approximately 50% of PTC cells (×10). (**E**) Almost all PTC cells showed moderate to intensive positivity for p53 (×40). (**F**) Weak to strong expression for p53 was detected in anaplastic cells (×40).

After applying immunohistochemical analyses, diffuse positivity was obtained for thyroglobulin TTF1, PAX8, and p53 in part of the tumor with PTC morphology. The anaplastic component of the tumor displayed a loss of expression for thyroglobulin and TTF1 while still maintaining positivity for PAX8 and aberrant positivity for p53. Both components showed high proliferative activity, with expression for Ki67 being more than 80% in the anaplastic component. The immunohistochemical analysis of the neck metastasis is shown in Figure 5. The definitive pathohistological diagnosis of the neck tumor was ATC arising from metastatic PTC with regressed primary PTC in the thyroid.

Additionally, tissue samples from both the metastasis and primary carcinoma were examined for the *BRAF* V600E mutation using the RealLine BRAF Detect-V600E Kit (BIORON Diagnostics GmbH, Römerberg, Germany) following the manufacturer’s instructions. The analysis was performed on the QuantStudio 5 real-time PCR system from Applied Biosystems, Thermofisher. A *BRAF* V600E mutation was detected in both samples.

Following the surgery, the patient was discharged from the clinic in good general condition with an indication of local radiotherapy. However, at the first check-up after one month, tumor bulging and necrosis in the area of the surgical incision were observed, indicating a progression of the disease. The patient received only substitution and symptomatic therapy. Two months after surgery, the patient died due to airway obstruction.

## 3. Discussion with Review of the Literature

Most s-PTCs are generally indolent, slow-growing, low-risk malignancies with a favorable outcome. Due to their indolence, the management option of active patient surveillance is preferred over immediate surgery [5,7,14]. Active surveillance studies show that 57–67.7% of s-PTCs do not change in size, while rapid tumor growth and novel lymph node metastasis develop in 17.2% and 3.8% of patients, respectively [15,16,17]. Adverse outcomes, such as anaplastic transformation, are exceedingly rare [10]. The findings from various studies indicate that s-PTM regression was identified in 4% to 17% of the cases [15,16]. STR is a well-known phenomenon that has been discussed in the literature for centuries. However, the exact mechanisms involved in tumor STR remain unclear. The current understanding suggests that antitumor immune responses play a significant role and can be triggered by factors such as inflammation, infection, fever, autoimmunity, biopsy procedures, and disruptions in the tumor microenvironment [1,2,18]. As results from active surveillance have shown, regression of s-PTC is not rare [17]. The reason for s-PTC regression is unknown. It may be related to the specific nature of s-PTC, which has limited growth potential, and the shrinkage of the tumor results from its senescence, followed by a reduction in tumor vascularity [7,17,19,20]. In cases of s-PTC, where an FNA biopsy was performed, its effect on regression cannot be ruled out [17]. Regression of PTC coinciding with Hashimoto’s thyroiditis, as in our case, is sporadically reported in the literature [4,21]. However, a study by Di Pasquale et al. noted pathological features of STR, including complete obliteration of the tumor by fibrosis with a significant reduction in the number of tumor cells in 26% of PTC cases coexisting with Hashimoto’s thyroiditis [22]. It is well established that autoimmunity in Hashimoto’s thyroiditis targets normal thyroid cells through both humoral and cytotoxic mechanisms. It can be suggested that this autoimmune response to healthy thyroid cells may also impact tumor cells, leading to restricted growth or even inducing their destruction [23,24].

The occurrence of metastases in the lymph nodes of s-PTCs in regression is rare and may represent the only clinical manifestation of these tumors [4]. Multiple metastases, especially those of larger sizes, can significantly influence the clinical course of the disease, making its biological behavior more similar to that of PTCs larger than 10 mm [15]. Morphological features of lymph node metastasis through the appearance of aggressive subtypes of PTC, such as tall cell and hobnail subtypes, may significantly affect clinical courses. Furthermore, the dedifferentiation of s-PTC lymph node metastasis or its transformation in ATC is associated with a rare occurrence of deadly outcomes [25,26].

Among the published cases, we identified 29 instances of PTC exhibiting anaplastic transformation in neck lymph nodes, as detailed in Table 1 [9,11,25,27,28,29,30,31,32,33,34,35,36,37,38,39]. Most of these cases, including our own, involved elderly patients [9,25,27,28,30,31,32,33,34,35,36,37,38,39]. Notably, even in the case reported by Park JH. et al., a young man aged 30 experienced an anaplastic transformation eight years after his diagnosis of PTC [29]. This agrees with the fact that the biological characteristics of ATC develop over a more extended period through the appearance of additional genetic and epigenetic changes in well-differentiated thyroid carcinomas [6].

Anaplastic transformation of s-PTC metastases has been reported in four cases, all of which represented the initial presentation of the disease [9,11,33,35]. In situations where the primary thyroid tumor is not clinically apparent, as in our case, additional clinical examinations, along with pathohistological and immunohistochemical analysis of biopsy or surgical specimens, are essential for tumor diagnostics and determining its primary origin. Distinguishing between lateral neck metastasis of unidentifiable primary thyroid carcinoma and primary thyroid carcinoma that originates from ectopic thyroid tissue also presents a significant diagnostic challenge. Morphology alone is insufficient to distinguish between these entities; thus, the regressed s-PTC may be incidental, and anaplastic transformation in the primary PTC of ectopic thyroid tissue cannot be ruled out. Molecular analysis of the metastasis and s-PTC may be the only option to resolve this conundrum [40]. Two studies performed a molecular analysis of major driver genes in all 29 reported cases of PTC with anaplastic transformation of lymph node metastasis [25,34]. Vishwanath et al. detected the *BRAF* V600E mutation in neck metastasis [34]. In the study by Odate et al., eight out of nine cases disclosed the *BRAF* V600E mutation along with *TERT* promoter mutation in all cases, both in the primary tumors and metastatic lymph nodes. The same study obtained similar results for a group of recurrent PTCs without anaplastic transformation, indicating that only a subset of recurrent PTCs with *TERT* promoter mutation are prone to undergo anaplastic transformation in nodal recurrence [25]. The same authors analyzed the presence of *p53* mutation through aberrant immunohistochemical positivity for p53. Unlike *TERT* promoter mutation, aberrant p53 expression was detected only in ATC and one of three hobnail-subtype PTCs [25]. We could not perform p53 analysis on the primary tumor because the remaining tissue lacked tumor representativeness. However, p53 positivity was observed in both components of the metastasis, which supports the evidence of *p53* gene mutation’s role in anaplastic transformation [5,6,41]. In addition to immunohistochemical analysis for p53, we performed molecular testing only for the *BRAF* V600E mutation, which is the main disadvantage of this work. Nevertheless, the *BRAF* V600E mutation in both specimens supports the same clonal origin of the tumors. Taken together, the final diagnosis in our case was based on suggestions of a recent study, which indicated that a diagnosis of metastatic thyroid carcinoma is more likely over carcinoma of ectopic thyroid tissue, even in cases where a primary thyroid tumor is suspected to be regressed and/or when multiple lymph node metastases accompany a lateral neck mass [42]. This approach was also supported by the research conducted by Xu B. et al. This study indicated that aggressive histology in metastatic thyroid carcinoma could appear even in patients with no identified primary thyroid tumor. The authors proposed that some thyroid carcinomas may stay undetected after whole-thyroid processing due to their small size or complete STR [43].

Most of the reported PTC cases with anaplastic lymph node metastasis transformation were of the classic subtype [9,25,27,31,32,33,35,36,39]. In a series of cases by Odate T. et al., the significance of solid/insular and hobnail patterns in recurrent metastases, with their subsequent anaplastic transformation, was highlighted [25]. Similarly, in the report by Salh AM et al. [38], as in our case, the well-differentiated component also exhibited a loss of cellular polarity and cohesiveness. These findings support earlier conclusions that the hobnail pattern observed in PTC, whether in primary tumors or nodal metastases, represents a high-grade dedifferentiation step that can lead to subsequent anaplastic transformation [41].

In cases of dedifferentiation of metastatic PTC, the outcomes are unfavorable and align with the prognosis typically observed in patients diagnosed with ATC. Early detection of ATC in either the primary tumor or its metastases facilitates prompt surgical intervention. Timely curative surgery can result in a better prognosis, as noted in a study by Ito et al. [9]. However, postoperative treatments such as locoregional radiotherapy and chemotherapy do not significantly enhance the prognosis of the disease. Encouraging results have been found with targeted therapy application. Namely, Vishwanath et al. reported that combining a BRAF inhibitor with an MEK inhibitor demonstrated remarkable efficacy, significantly reducing the size of *BRAF* V600E-mutated ATC [34]. These findings align with the current recommendation for testing ATC for the *BRAF* V600E mutation since an aforementioned combination of inhibitors was found to be active against *BRAF* V600E-mutated ATC [6,34].

## 4. Conclusions

Anaplastic transformation of s-PTC metastases, specifically those with low-risk features, is exceptionally rare. The synchronous co-occurrence of incidental primary thyroid s-PTC and the anaplastic transformation of PTC originating from ectopic cervical thyroid tissue must be considered diagnostically. This case underscores the clinical importance of regular follow-up of the thyroid gland and neck lymph nodes for patients under active surveillance for s-PTC, including those with small calcified tumor foci.

## Figures and Tables

**Table 1:** Summary of case reports of PTC with anaplastic transformation into neck lymph node metastasis.

No.	Study (Year)	Age (Years)/Gender	Size (mm)	PTC Subtype	Time to AT (Months)	Treatment	DOD (Months)
1.	Sato K et al. (2006) [27]	77/M	20	Classic	0	NA	6
2.	Ito Y et al. (2008) [9]	64/M	35	Classic	46	Ro	5
3.	Ito Y et al. (2008) [9]	80/F	25	Classic	0	NA	Alive 85 months after surgery
4.	Ito Y et al. (2008) [9]	51/F	25	Classic	266	Ch	63
5.	Ito Y et al. (2008) [9]	79/M	13	Classic	74	NA	11
6.	Ito Y et al. (2008) [9]	77/F	4	Classic	0	Ro	Alive 6 m after surgery
7.	Awan L et al. (2013) [28]	50/F	NA	NA	48	Ro+ Ch	NA
8.	Deutschmann M et al. (2013) [11]	60/M	5	NA	0	Rai+Ro	NA
9.	Park JH et al. (2014) [29]	31/M	40	DSS	96	NA	4
10.	Gunnarsdottir AB et al. (2018) [30]	79/M	NA	NA	0	Ro	3
11.	Cinamon U et al. (2020) [31]	68/M	15	Classic	96	Ch	5
12.	Song S et al. (2020) [32]	85/M	18	Classic	0	Not taken	2.5
13.	Tallab R et al. (2020) [33]	84/M	5	Classic	0	Not taken	5
14.	Vishwanath V et al. (2021) [34]	60/F	NA	Foll	0	TT	Alive
15.	Momin YA et al. (2021) [35]	75/F	5	Classic	0	NA	NA
16.	Odate T et al. (2021) [25] 10 cases	2/M; 8/F	33.4 ± 17.4	8 Classic; 1 Foll; 1 Tall	106 [6, 437] *	4 Rai; 6 NA	NA
17.	Fukuda Y et al. (2022) [36]	67/F	NA	Classic	0	Ro	1.5
18.	Prabhu SAC et al. (2022) [37]	72/M	NA	NA	60	Ro	NA
19.	Salh AM et al. (2022) [38]	91/F	NA	Hobnail	132	Ro	NA
20.	Lyu YS et al. (2024) [39]	63/M	NA	Classic	2	Ro	2

PTC—papillary thyroid carcinoma; AT—anaplastic transformation; DOD—died of disease; NA—not available; Ro—radiotherapy; Ch—chemotherapy; Rai—radioactive iodine therapy; TT—targeted therapy with the BRAF inhibitor; Foll—follicular subtype of PTC; Tall—tall cell subtype of PTC; DSS—diffuse sclerosing subtype of PTC. * Median [range].

## Data Availability

All relevant data are reported in the article.
